# Supplementary material for: The mediating role of isolation and marginalization in the effect of trust in school principals on organizational commitment: physical education and sports teachers
Source: BMC Psychol. 2025 Oct 16;13:1134. doi: 10.1186/s40359-025-03504-3 (PMC12529786; doi:10.1186/s40359-025-03504-3)
Supplement: Supplementary file 1 — Supplementary Material 1. [file 40359_2025_3504_MOESM1_ESM.docx]

**Full Article Title:** The Mediating Role of Isolation and Marginalization in the Effect of Trust in School Principals on Organizational Commitment: Physical Education and Sports Teachers

**Each Author’s Complete Name and Institutional Affiliation(s):**

Emrah SEÇER, PhD^1^, Ahmet Yavuz MALLI, PhD^2^, Hasan Buğra EKİNCİ PhD^3^, Oğuz Kaan ESENTÜRK, PhD^4^

^1^Assistant Professor, Erzincan Binali Yıldırım University, Faculty of Sport Sciences, Erzincan, Turkey, Orcid: https://orcid.org/0000-0002-6683-680X, emrah.secer10@gmail.com

^2^Assistant Professor, Erzincan Binali Yıldırım University, Faculty of Sport Sciences, Erzincan, Turkey, Orcid: https://orcid.org/0000-0001-6478-4893, ahmet.malli@erzincan.edu.tr

^3^Assistant Professor, Erzincan Binali Yıldırım University, Faculty of Sport Sciences, Erzincan, Turkey, Orcid: https://orcid.org/0000-0002-3883-2983, hasan.ekinci@erzincan.edu.tr

^4^Associate Professor, Erzincan Binali Yıldırım University, Faculty of Sport Sciences, Erzincan, Turkey, Orcid: https://orcid.org/0000-0002-0566-838X, esenturk954@gmail.com

**Corresponder Author:**

Emrah SEÇER,PhD

Asst. Prof., Faculty of Sport Sciences

Adress: Erzincan Binali Yıldırım University, Erzincan,24100, Turkey

Mail: emrahsecer10@gmail.com

Phone: +905455531753
