# Supplementary material for: The mediating role of isolation and marginalization in the effect of trust in school principals on organizational commitment: physical education and sports teachers
Source: BMC Psychol. 2025 Oct 16;13:1134. doi: 10.1186/s40359-025-03504-3 (PMC12529786; doi:10.1186/s40359-025-03504-3)
Supplement: Supplementary file 3 — Supplementary Material 3. [file 40359_2025_3504_MOESM3_ESM.docx]

**The Mediating Role of** **Isolation and Marginalization in the Effect of Trust in School Principals on Organizational Commitment: Physical Education and Sports Teachers**

Emrah SEÇER^1^, Ahmet Yavuz MALLI^1^, Hasan Buğra EKİNCİ^1^, Oğuz Kaan ESENTÜRK^1^

^1^Assistant Professor, Erzincan Binali Yıldırım University, Faculty of Sport Sciences, Erzincan, Turkey, Orcid: https://orcid.org/0000-0002-6683-680X, emrahsecer10@gmail.com

^1^Assistant Professor, Erzincan Binali Yıldırım University, Faculty of Sport Sciences, Erzincan, Turkey, Orcid: https://orcid.org/0000-0001-6478-4893, ahmet.malli@erzincan.edu.tr

^1^Assistant Professor, Erzincan Binali Yıldırım University, Faculty of Sport Sciences, Erzincan, Turkey, Orcid: https://orcid.org/0000-0002-3883-2983, hasan.ekinci@erzincan.edu.tr

^1^Associate Professor, Erzincan Binali Yıldırım University, Faculty of Sport Sciences, Erzincan, Turkey, Orcid: https://orcid.org/0000-0002-0566-838X, esenturk954@gmail.com

**Corresponder Author:**

Emrah SEÇER, Asst. Prof., Faculty of Sport Sciences, Adress: Erzincan Binali Yıldırım University, Erzincan, 24100, Turkey^,^ Mail: [emrahsecer10@gmail.com](mailto:emrahsecer10@gmail.com)^,^ Phone: +905455531753

**Purpose:** This study examines how teachers' perceptions of their principal's trust in them are associated with their organizational commitment, with a particular focus on the mediating role of marginalization and isolation.

**Methodology:** Data were collected from 247 physical education and sports teachers using validated instruments measuring perceived principal trust, organizational commitment, and marginalization and isolation. Confirmatory factor analysis and bootstrap mediation analyses were conducted.

**Results:** The findings revealed that perceived principal trust was positively associated with organizational commitment and negatively associated with marginalization and isolation. Higher levels of perceived trust corresponded with lower feelings of marginalization and isolation, which in turn were linked to more substantial organizational commitment. The indirect effect of marginalization and isolation was statistically significant, providing support for the mediation hypothesis.

**Conclusion:** Acknowledging the study's cross-sectional design, the results suggest that leadership practices aimed at enhancing teachers' perception of being trusted are crucial for mitigating feelings of marginalization and fostering organizational commitment. Practical implications involve promoting leadership behaviors that not only build trust but also actively and clearly signal it, thereby creating a more inclusive and supportive school environment where all teachers feel valued.

**Keywords:** Physical education teachers, Organizational commitment, Perception of trust, Marginalization and isolation

**Introductıon**

The concept of trust is a phenomenon that has been extensively discussed in social sciences and analyzed from various angles. Trust is also an important element in educational institutions. The school should create an environment where trust is provided and felt by students, teachers, and other stakeholders. Trust is essential for physical education teachers, who often face skepticism or undervaluation from administrators and colleagues because of perceptions regarding the lower academic priority of their subject [[1](#Cengiz)]. Research shows that a strong principal-teacher relationship positively affects teachers' motivation, job performance, and loyalty to the institution. It is stated that variables such as success, trust, and respect are important among teachers' expectations from school climate and administrators [[2](#Quines), [3](#Ukuşlu)]. These factors increase teachers' commitment and satisfaction [[4](#Lenz)], increase their well-being [[5](#Zheng)], and support their engagement. Thus, committed teachers can work effectively in a positive school climate [[6](#Martinsone)]. Organizational commitment is the sense of belonging that an individual feels towards the organization, the adoption of the goals and values of the organization and the willingness to stay in the organization in this direction. Organizational commitment is also seen as an effective factor in work discipline and health [[7](#Sarumaha)]. Teachers carry out a professional education process by establishing close relationships with other stakeholders in schools and strive to achieve the goals of the organization by feeling a high level of commitment [[8](#Ramli)]. However, the low level of commitment of the employees in the organization can negatively affect the efforts to achieve the goals and cause the working environment to become inefficient [[9](#Yuwono)]. This situation may lead to a decrease in the quality of education [[10](#AlRefaei)] and may also negatively affect the overall success of the organization. In this context, considering that the lack of organizational commitment affects not only the general working environment but also teachers' professional identities and motivation for educational processes, the marginalization and isolation behaviors that physical education teachers are exposed to should be considered as a special reflection of these effects. Marginalization and isolation refers to the process by which individuals or groups are pushed out of the social structure, away from social interaction, detached from the center and away from social integration. Physical education teachers often experience marginalization and isolation due to perceptions among administrators and colleagues that their subject lacks academic rigor or holds lower priority compared to other subjects [[11](#Richards11)]. This marginalization is manifested through limited access to professional resources, exclusion from decision-making processes, and reduced professional interaction with other faculty members [[12](#Gaudreault)]. Consequently, physical education teachers frequently encounter feelings of professional frustration, isolation, decreased job satisfaction, and reduced organizational commitment, negatively impacting their motivation and overall effectiveness in educational environments [[11](#Richards11), [12](#Gaudreault)]. Physical education teachers experience higher levels of isolation and marginalization compared to teachers in other subject areas [[11](#Richards11), [13](#Shoval)]. Teacher isolation is based on the pioneering work of Flinders [[14](#Flinders)] and Lortie [[15](#Lortie)]. In the context of teacher isolation, Flinders [[14](#Flinders)] argued that isolation is a common feature of professional life in schools and that it is a potential barrier to the implementation of reform initiatives because it limits opportunities for professional development. Despite considerable research on trust and organizational commitment, studies explicitly focusing on the marginalization and isolation specifically faced by physical education teachers remain limited. This represents a critical gap that this study seeks to address [[16](#Gaudreault16)]. This represents a significant gap that needs to be addressed [[17](#Walters)].

Addressing this gap is particularly important because the marginalization of physical education teachers not only threatens their individual well-being and professional engagement, but also risks undermining the delivery and perceived legitimacy of physical education as an essential component of holistic education. When these teachers are excluded from core school processes, it diminishes interdisciplinary collaboration, weakens the integration of physical activity into the broader curriculum, and reinforces systemic inequalities in educational priorities. By empirically examining how trust relates to organizational commitment through marginalization and isolation, this study contributes both to theory and to practical efforts to build more inclusive, equitable school environments where all educators—regardless of subject area—feel valued and supported.

This study is theoretically grounded in Social Exchange Theory (SET) and the Psychological Exclusion Model. Consistent with SET, our work is based on the premise that the quality of relationships within a school, including formal and informal group dynamics [[18](#Gök)], is foundational to organizational outcomes. More specifically, the literature identifies interpersonal trust between teachers and school leaders as a "fundamental pillar" of this dynamic [[19](#Razeto)]. This trust functions as a critical form of organizational support and a socio-emotional resource [[20](#Dursun)] that, in turn, has significant predictive power over teachers' organizational commitment [[21](#Babaoglan)].

The Psychological Exclusion Model provides the theoretical lens for this dynamic. It posits that the need for social inclusion is not merely a preference but a fundamental aspect of human psychology, rooted in the evolutionary development of our relational and social brain [[22](#Fante)]. When this core need is unmet through perceived marginalization, it can damage an individual’s sense of belonging and organizational integration. This is not just an internal state; research shows that a lack of social and psychological inclusion is directly linked to negative student outcomes [[23](#Munguia)], highlighting its critical importance within educational psychology [[24](#Hutchison)].

Although prior research has explored the links between trust and commitment in schools, and even within specific sports contexts [[25](#Tsai)], and while studies have indeed confirmed a relationship between marginalization and commitment among physical education teachers [[26](#Ulaş)], limited empirical attention has been given to the specific role of trust as a protective factor that mediates this negative dynamic. This represents a critical gap, as this group remains structurally and symbolically undervalued in many educational systems.

This study aims to fill this gap by integrating these theories into a model that explains how school principals' trust is associated with lower levels of marginalization and isolation, and how these factors in turn are related to PE teachers' affective organizational commitment. The study’s unique contribution lies in (1) the explicit conceptual linkage between interpersonal trust and workplace exclusion, (2) the empirical modeling of marginalization and isolation as mediators, and (3) its implementation in a collectivist, culturally specific educational context—Turkey—where relational dynamics play a key role in organizational behavior.

It can be stated that employees with high organizational commitment prioritize trust, respect, understanding, and support in their relationships in the work environment and are not marginalized or isolated. In this context, this study aims to examine the effect of school principals' trust in teachers on teachers' organizational commitment and the mediating role of marginalization and isolation in this effect. In this study, trust refers to the degree to which school principals perceive teachers as competent, honest, and trustworthy professionals and is considered a unidimensional construct. Organizational commitment is defined solely by the affective commitment dimension, which reflects teachers' emotional attachment to their schools and sense of institutional belonging. Marginalization refers to a teacher's perception that their branch or professional role is excluded from basic education processes or is not adequately valued. Isolation, on the other hand, describes a teacher's subjective experience of social and professional disconnection from colleagues. Analyzing the relationship between these variables is important in terms of providing a road map for the administrators of educational institutions. This situation emphasizes the importance of the study in terms of its contribution to the literature as well as its practical implications.

**Literature review**

**Theoretical support**

The theoretical basis of this study is based on the principle that an employee's perception of their work environment is a stronger determinant of behavior than objective reality itself. Therefore, the fundamental structure of this research is not managers' trust in teachers, but rather teachers' perceived trust in managers. This concept, hereafter referred to as perceived trust in managers, represents a teacher's subjective interpretation and belief regarding the extent to which they view their manager as competent, honest, and trustworthy. This perception serves as a critical psychological and social resource. This study adopts a multi-faceted approach to explain how perceived trust affects organizational commitment, particularly through its impact on teachers' feelings of othering and isolation.

Social Exchange Theory (SET) provides a fundamental perspective for understanding this dynamic [[27](#Nazir)]. SET assumes that social relationships are based on a process of exchange in which individuals weigh costs and rewards. Within this framework, perceived principal trust is a crucial socio-emotional reward for teachers. When teachers perceive that they are trusted, they interpret this as a signal of respect, value, and inclusion; a type of relational investment from their leaders. According to the norm of reciprocity, one of the fundamental principles of SET, this perceived investment creates a sense of obligation in the teacher to reciprocate. This reciprocity manifests itself in positive work attitudes and behaviors, notably stronger emotional organizational commitment [[28](#Chow)]. This is consistent with Leader-Member Exchange (LME) theory, which posits that high-quality leader-follower relationships are characterized by mutual trust and support [[29](#Graen)]. Perceived manager trust is a strong indicator of high-quality LMI. It places the teacher within the leader's “in-group,” thereby acting as a buffer against the social threats of marginalization and isolation.

The Psychological Exclusion Model further clarifies the mechanism by which perceived lack of trust leads to adverse outcomes. This model posits that the need to belong is a fundamental human motivation [[30](#Williams)]. A low perception of trust in a principal functions as a strong signal of social exclusion, threatening this basic need. It fosters feelings of being marginalized and isolated by giving the teacher the impression that they are not a valued member of the school community. On the other hand, a perceived high level of trust acts as an antidote to this threat, strengthening the teacher's sense of belonging and psychological security.

Theories supporting this view, such as Maslow's Hierarchy of Needs and Empowerment Theory, highlight how perceived trust operates. From a Maslowian perspective, feeling trusted by a principal directly satisfies higher-level needs such as “belonging” and “respect” [[31](#Yazgı)]. When teachers feel that their competence is recognized and valued, they are more likely to progress toward self-actualization. Similarly, Empowerment Theory explains how specific leadership behaviors create this critical perception [[32](#Somech)]. When principals involve teachers in decision-making processes, grant them autonomy, and value their ideas, they not only delegate authority but also actively demonstrate trust [[33](#Dong)]. These empowering practices make teachers feel valued and competent, which directly reduces their perceptions of marginalization and, consistent with SET, enhances their motivation and commitment to the organization [[34](#Bellibas)].

These theoretical frameworks model perceived principal trust as a socio-emotional resource that, when received by a teacher, initiates positive social interaction. This perception enhances psychological safety, fulfills basic needs such as belonging and esteem, and ultimately fosters a more substantial organizational commitment by mitigating the psychological threats of marginalization and isolation. This integrated model provides a solid foundation for our hypotheses.

**Hypotheses development**

Previous research has investigated the marginalization and isolation experiences of physical education teachers extensively [[35](#Organista), [36](#Sakallı36)]. However, there is a notable lack of studies that explicitly address how marginalization and isolation mediate the relationship between teachers' perceptions of their principal's trust and their subsequent organizational commitment. Addressing this gap is crucial for understanding how the feeling of being trusted relates to organizational commitment, specifically through the psychological processes of reduced marginalization and isolation, thereby providing a stronger theoretical and practical foundation for our hypotheses.

**The Role of Perceived Principal Trust in Fostering Organizational Commitment**

Throughout history, all societies have continuously strived to have the best education systems, viewing education as an indispensable element of development. In a globalizing world, it is emphasized that education systems and institutions that fail to adapt to the requirements of the age are gradually losing their importance [[37](#Canlı37)]. Educational institutions play a critical role in shaping the future of societies, performing a fundamental function in human resource development, cultural transmission, and social interaction processes [[38](#Sevinç)]. In this context, schools, as the fundamental structures that educate future generations, are considered the most central element among educational institutions.

Schools are organized structures that bring together various stakeholders, including administrators, teachers, and students, to achieve educational goals and objectives. Ensuring institutional integrity and cooperation requires stakeholders who perform their duties effectively and leadership figures with strong communication skills. At this point, school administrators hold a decisive position in shaping the school climate. Embracing innovation [[39](#Nacar)], setting institutional goals, and creating positive learning environments [[40](#Sarwar)] are the main factors that strengthen their role. School principals, as primary leaders, utilize various management skills such as encouraging teamwork, boosting motivation, and directing organizational goals to improve the quality of the learning process [[41](#Leithwood)]. Leadership characteristics such as creating a clear vision, establishing strong communication, and ensuring social justice and fairness are frequently highlighted in the literature as critical elements of effective school leadership [[42](#Akbaşlı)].

However, the impact of these leadership actions is determined more by how teachers perceive them than by the behaviors exhibited by principals. A school principal's fundamental responsibility is to establish an environment where teachers feel trusted, supported, and psychologically safe. Research shows that teachers who report feeling unsupported or insecure experience adverse effects on their motivation and performance [[43](#Musah)]. Therefore, principals must exhibit behaviors that foster teachers' perceptions of trust and a sense of belonging. This subjective bond with the school is based on the teacher perceiving themselves as a valued, respected, and reliable part of the institution.

In this context, the concept of organizational commitment takes center stage. Organizational commitment is a crucial factor that solidifies the psychological bond teachers establish with their institutions and reflects their level of identification and dedication to their work [[44](#Kırkıç)]. Teachers' identification with the school's goals, their emotional attachment to the institution, and their sense of belonging integrated with their perceptions of trust are at the core of this commitment [[5](#Zheng)]. Research shows that teachers with a strong sense of belonging are more willing to invest their time and energy in the institution [[45](#Yao)]. Furthermore, the perception of trust has a positive impact on other critical psychological processes, such as feeling appreciated, valued, and treated fairly [[46](#Liu)].

Based on this theoretical framework, the following hypothesis is proposed:

H1: Teachers' perceptions of their school principals' trust in them are positively related to their organizational commitment.

**The Mediating Role of Marginalization and Isolation**

H1 suggests a direct relationship between perceived trust and organizational commitment, while the existing literature indicates that this relationship may have a more complex structure, particularly for vulnerable groups of teachers. Professional Socialization Theory offers a critical framework for understanding this complexity by highlighting that teachers' professional identities are shaped through social experiences within the school culture [[47](#Parker)]. According to this perspective, physical education teachers are often perceived as having a marginal status within educational institutions; academically, they are seen as less valuable than teachers in other subjects. This situation can lead them to feel excluded or isolated from the school's central activities and peer networks [[48](#Stroot)]. These experiences of exclusion create uncertainty in teachers' professional attitudes, negatively affecting their performance and weakening their commitment to the institution [[49](#Şenel49)].

In this context, the perception of trust in principals emerges as a critical protective factor for teachers. Perceived trust acts as a powerful antidote to feelings of exclusion and isolation. The concept of organizational justice plays a vital role in explaining this process. Administrators' adoption of fair, transparent, and equitable practices sends a strong message to teachers—regardless of subject area—that they are a valuable and indispensable part of the institution [[50](#Chen)]. When teachers experience these fair practices, they interpret them as concrete indicators that they are trusted and respected. Thus, the perception of justice and trust reinforces feelings of belonging and being valued, reducing the psychological threats that marginalization and isolation can cause.

Therefore, it is argued that the effect of perceived principal trust on organizational commitment is not only direct but also indirect, through its function of reducing teachers' experiences of marginalization and isolation. This logic is consistent with Professional Socialization Theory, which defines marginalization as a lack of socialization that weakens commitment, particularly in the context of teachers who are assigned low value. Based on this mediating mechanism, the following hypothesis is proposed:

H2: Teachers' experiences of marginalization and isolation mediate the relationship between their perceptions of school principals' trust in them and their organizational commitment.

**Research Methodology**

**Research Model**

This study investigates the effect of school principals' trust in physical education and sports teachers on their commitment to the organization. In addition, the mediating role of marginalization and isolation in this effect is examined. From this point of view, the research was conducted in the relational survey model, which is one of the quantitative research methods used to determine the relationships between two or more variables [[51](#Christensen)]. The theoretical model to be tested in the research is presented in [Figure 1.](#Fig1)

Marginalization and Isolation

(-)

(-)

a*b (+) = H2

a (-)

b (-)

c (+) = H1

School Principals' Trust in Teachers

Organizational Commitment

**Fig. 1** Research Model

Due to the complexity of the relationships between variables in social research, it may be insufficient to identify a simple or direct relationship. How the relationship between two variables emerges through a connection mechanism (mediation) is important for understanding the social reality. It is stated that the way to increase the existing knowledge and to add a unique value to the related theory and relationship pattern can be realized by testing the mediating effects [[52](#Gürbüz)].

**Participants and Procedure**

The research group was determined using a non-probability convenience sampling method [[53](#Cohen)]. An online survey link was distributed through national physical education teacher associations, professional online forums, and social media groups dedicated to physical education and sports teachers in Turkey. The invitation specified that the study was intended for currently employed physical education and sports teachers working in public schools. Participation was entirely voluntary and anonymous. This recruitment strategy aimed to reach a broad and accessible sample of the target population. G*Power analysis was conducted to determine the required sample size, indicating that a minimum of 193 participants were needed to achieve 80% statistical power with an alpha of .05 and a small to medium effect size (correlation = 0.20) [[54](#Richard54)]. The final sample exceeded this requirement, consisting of 247 active physical education teachers. The sample included 176 males (71.3%) with a mean age of 37.08 (SD = 8.87) and 71 females (28.7%) with a mean age of 32.27 (SD = 8.32).

**Data Collection Tools**

**Scale for Determining School Principals' Trust in Teachers-Teacher Form:** The scale developed by Canlı et al. [[55](#Canlı55)] consists of 18 items and 4 sub-dimensions (competence, trustworthiness, openness, helpfulness). The 5-point Likert-type scale is evaluated in the range of "1=Never, 5=Always". Items in the credibility dimension of the scale are reverse-coded. During the development phase of the scale, the Cronbach alpha *(a)* internal consistency coefficient was calculated as .916 for the competence dimension, 861 for the trustworthiness dimension, .793 for the openness dimension, and .755 for the helpfulness dimension. The model fit values of the scale were found as (χ2 /df = 3.801, GFI = .92, AGFI = .89, CFI = .94, RMSEA = .07, SRMR=.08). In this study, the factor structure of the scale consisting of 4 sub-dimensions and 18 items were tested with Level 2 CFA. As a result of the normal distribution of the data, the Maximum Likelihood calculation method was used. As a result of CFA, the goodness of fit values accepted in the literature could not be reached. Therefore, 3 modifications (e11🡪 e12, e5🡪 e6, e17🡪 e18) were made by examining the modification indices. In this context, AVE and CR values were calculated. AVE and CR values were found to be 0.60 and 0.96, respectively.

**Organizational Commitment Scale for Teachers:** The 5-point Likert-type scale designed by Üstüner [[56](#Üstüner)] to measure teachers' organizational commitment consists of 17 items and one factor. Cronbach alpha *(a)* internal consistency coefficient was calculated as .96 during the development of the scale. The model fit values of the scale were found as r (χ2 /df = 2.86, GFI = .89, AGFI = .85, CFI = .95, RMSEA = .078, SRMR=.035). In this study, the factor structure of the scale consisting of a single dimension and 17 items was tested with Level 1 CFA. As a result of the normal distribution of the data, the Maximum Likelihood calculation method was used. As a result of CFA, the goodness of fit values accepted in the literature could not be reached. Therefore, 3 modifications (e1🡪 e2, e3🡪 e4, e14🡪 e15) were made by examining the modification indices. In this context, AVE and CR values were calculated. AVE and CR values were found to be 0.60 and 0.96, respectively.

**Physical Education - Marginalization and Isolation Scale:** The scale developed by Gaudreault, Richards and Woods [[12](#Gaudreault)] was adapted into Turkish by Şenel et al. [[49](#Şenel49)]. The scale has two dimensions (marginalization, isolation), 10 items, and a 7-point Likert-type scale. The Cronbach alpha *(a)* internal consistency coefficient during the adaptation phase of the scale was calculated as .74 for the marginalization sub-dimension, .70 for the isolation sub-dimension, and .96 for the overall scale. The model fit values of the scale were found as r (χ2 /df = 2.59, CFI=.93, RMSEA = .07, SRMR=.07). Within the scope of this study, the factor structure of the scale consisting of 2 factors and 10 items was tested with Level 2 CFA. As a result of the normal distribution of the data, the Maximum Likelihood calculation method was used. As a result of CFA, the goodness of fit values accepted in the literature could not be reached. Therefore, 3 modifications (e1🡪 e2, e8🡪 e9, e1🡪 e3) were made by examining the modification indices. In this context, AVE and CR values were calculated. AVE and CR values were found to be 0.52 and 0.84, respectively.

In all CFA applications, modification indices suggested correlations between residual terms within the same subdimension (e.g., e11 → e12). These adjustments were theoretically justified based on semantic proximity and shared item context. According to Kline (2023) [[57](#Kline)], correlating error terms among conceptually similar items within the same latent construct is an acceptable practice when it improves model fit without distorting theoretical integrity. These decisions were made conservatively, with no cross-loading or item removal.

**Data Collection**

Data were collected using an online survey created with Google Forms. The survey link was distributed through national physical education teacher associations, professional online forums, and social media groups. The initial page of the survey presented an informed consent form, which outlined the study's objectives and assured participants of their anonymity and confidentiality. It stated that participation was voluntary and could be terminated at any time. After providing consent, participants proceeded to the demographic questions and the measurement scales. The estimated completion time for the survey was approximately 10 minutes. All procedures were conducted after receiving formal ethical approval.

**Data Analysis**

Before analyzing the data, outliers and missing values were examined using IBM SPSS 25, and since there were no outliers, the analyses were conducted on 247 teachers. The suitability of the data for normal distribution was examined and the relationships between the independent, dependent, and mediating variables in the study were tested with Pearson correlation analysis. The suitability of the data in terms of normal distribution was analyzed in terms of Mahalanobis distances, Z values, skewness, and kurtosis values of the calculated scale scores. It is stated that if the skewness and kurtosis values are between +1 and -1, the data are normally distributed [[58](#Hair)]. In addition, it was determined that the Z values were inappropriate ranges (-3/+3). In addition, linear relationships between the variables were checked with the scatter diagram and it was seen that there was no deviation in the distribution. In addition, the tolerance and VIF values obtained from the data gave results confirming that there was no multicollinearity between independent variables (Tolerance > 0.2, VIF < 10). The average variance (AVE) and construct reliability (CR) values of the scales used in the study were calculated and it was determined that they met the criteria of AVE>.70, CR>.05 and AVE>CR. AMOS 21 program was used to test the factor structure of the measurement tools used in the study. After confirming the factor structure of the measurement instruments, regression analysis based on the Bootstrap method was applied to test the research hypotheses [[59](#Hayes59)]. The hypotheses were tested in line with model number 4 using Process Macro developed by Hayes [[59](#Hayes59)]. With Model 4, the mediating role of physical education-othering and isolation was examined. In the analyses, the Bootstrap technique and 5000 sample option were preferred [[60](#Hayes60)]. The selection of Model 4 in PROCESS macro is theoretically aligned with our study framework. As our hypotheses posit a simple indirect effect—where the relationship between teachers' perceived principal trust and organizational commitment operates through marginalization and isolation—this model offered the most parsimonious and theoretically coherent approach. We intentionally did not include moderation or serial mediation structures, as no interaction effects or sequential mediation pathways were hypothesized. The conceptual simplicity of Model 4 enhances interpretability and aligns well with prior literature in educational and organizational psychology. In mediation effect analyses conducted with the Bootstrap technique, the values in the 95% confidence interval obtained as a result of the analysis should not include the value 0 (zero) to support the research hypothesis [[59](#Hayes59), [61](#Preacher)].

**Results**

**Measurement Models and Reliability**

**Table 1:** Goodness of fit indices and threshold values used in structural equation modelling

| **Index** | **Good Fit** | **Acceptable** | **SPTT** | **OC** | **MI** |
| --- | --- | --- | --- | --- | --- |
| X^2^ /df | <3 | <3(X^2^ /df)<5 | 2.312 | 2.805 | 3.677 |
| GFI | >.95 | >.90 | .885 | .867 | .917 |
| CFI | >.95 | >.90 | .940 | .941 | .894 |
| RMSEA | <.95 | <.08 | .079 | .076 | .074 |
| Cronbach Alpha | - | - | .840 | .962 | .788 |

According to [Table 1](#Table1), the results of the confirmatory factor analyses indicated that all measurement models were compatible with the data and acceptable. This included the four-factor model of the scale used to assess teachers' perceptions of their principal's trust, the one-factor model of the organizational commitment scale, and the two-factor model of the marginalization and isolation scale. These results confirm the predicted theoretical constructs of the scales [[52](#Gürbüz), [62](#Byrne)]. While the initial analyses showed goodness-of-fit values that required improvement, modifications were made by associating the error terms of some items, as suggested by modification indices. With these adjustments, the fit indices for all scales were increased to acceptable levels, ensuring their construct validity. Furthermore, the Cronbach's Alpha reliability coefficients for all scales were determined to be relatively high, indicating strong internal consistency [[63](#Kalaycı)].

**Correlation Analysis and Descriptive Statistics**

[Table 2](#Table2) presents the correlations, descriptive statistics, and reliability values between the variables of the study, namely, teachers' perceptions of their principals' trust in them, their organizational commitment, and their levels of isolation and marginalization.

**Table 2:** Correlations between variables and descriptive analyses

| **Variables** | **1** | **2** | **3** | **X** | **Ss** | **Skewness** | **Kurtosis** |
| --- | --- | --- | --- | --- | --- | --- | --- |
| 1- School Principal's Trust in Teachers | 1 |  |  | 63.89 | 11.062 | -.662 | .532 |
| 2- Organizational Commitment | **.675**** | 1 |  | 66.77 | 14.242 | -.805 | .344 |
| 3- Marginalization-Isolation | **-.375**** | **-.393**** | 1 | 26.24 | 10.327 | .318 | -.716 |

***p<.05, **p<.01**

[Table 2](#Table2) shows that statistically significant relationships exist between teachers' perceptions of principal trust and the other variables. According to the results of the Pearson correlation analysis, a positive and moderately significant relationship was found between teachers' perceived principal trust and their organizational commitment (r = .675, p < .001). This finding supports the first hypothesis (H1) of the study. Furthermore, negative and moderately significant relationships were found between perceived principal trust and marginalization and isolation (r = -.375, p < .001) and between organizational commitment and marginalization and isolation (r = -.393, p < .001).

**Table 3:** Regression analysis results for mediation test (n=247)

|  |  |  |  | **Outcome Variables** | | |  |  |
| --- | --- | --- | --- | --- | --- | --- | --- | --- |
|  | **MI** | | | | **OC** | | | |
| **Forecast Variables** |  | *b* | *S.H.* | LLCI/ULCI |  | *b* | *S.H.* | LLCI/ULCI |
| **SPTT** | *a* | -.350** | .055 | -.4589/-.2411 | ***c^’^*** | .790** | .064 | .6643/.9172 |
| **MI** | *-* | - | - | - | *b* | -.224** | .068 | -.3596/-.0886 |
|  | *R^2^ =-.140* | | | | *R^2^* =.478 | | | |
|  | *F(*1;245)=40.0796; *p*<.001 | | | | *F*(2;244)=111.9431; *p*<.001 | | | |

*p<.05, **p<.01; S.H: Standard Error, (b): Unstandardized beta coefficients), SPTT: School Principals' Trust in Teachers, OC: Organizational Commitment, MI: Marginalization and Isolation, LLCI: Lower Level Confidence Interval, ULCI: Upper Level Confidence Interval

**Physical Education - Marginalization and Isolation**

b=.-.224, p<.01

a=-.350, p<.01

a*b = .078 (H2)

c=.869, p<.01 (H1)

**c**^’^ =.790, p<.01

**Organizational Commitment for Teachers**

**School Principals' Trust in Teachers**

Total Effect (c)= .869, p<.01

Direct Effect (**c^’^** )==.790, p<.01

Indirect Effect=.078; 95% LLCI=.0289, ULCI=.1410

(*b*): Unstandardized beta coefficients are reported.

**Fig. 2** Regression analysis results for mediation test

[Table 3](#Table3) and [Figure 2](#Fig2) present the results of the bootstrap mediation analysis, which tested the study's second hypothesis (H2). The findings reveal the pathways through which teachers' perception of their principal's trust influences organizational commitment. First, the analysis showed that perceived principal trust had a significant adverse effect on teachers' feelings of marginalization and isolation (b = -.350; p < .001, 95% CI [-.4589, -.2411]). Second, when controlling for the mediator, perceived principal trust remained a significant predictor of organizational commitment (b = .790; p < .001, 95% CI [.6643, .9172]), indicating a substantial direct effect. Furthermore, marginalization and isolation had a significant adverse impact on organizational commitment (b = -.224; p < .001, 95% CI [-.3596, -.0886]). Most importantly, the analysis confirmed a significant indirect effect. A positive relationship was observed between teachers' perception of their principal's trust and their organizational commitment. This relationship appears to be partially explained by the finding that higher perceived trust is associated with reduced feelings of marginalization and isolation. (Indirect Effect β = .078; p < .001; 95% CI [.0289, .1410]). This result provides strong support for Hypothesis 2. This finding suggests that the mediating role of reduced marginalization and isolation partially explains the positive relationship between perceived trust and organizational commitment.

**Discussion**

The findings of this study suggest that teachers' perceptions of their principals' trust in them are positively correlated with their organizational commitment. Furthermore, this relationship is partially mediated by teachers' experiences of being othered and isolated. The findings position perceived trust as a psychological resource that shapes the teacher's experience in the school environment and protects against the adverse effects of professional exclusion.

The results are strongly consistent with Social Exchange Theory (SET), which posits that positive social interactions support organizational commitment. This research brings a new perspective to the process by focusing on teachers' subjective perceptions rather than objective leadership characteristics. This emphasis on the teacher's perspective is consistent with recent studies highlighting the importance of this perspective in understanding the link between leadership and school performance [[64](#Savas), [65](#Shen)].

Within the SET framework, perceived principal trust is not a passive observation; it is an interpretation of a valuable socio-emotional reward for teachers. This study extends this understanding by framing trust as a psychological resource that buffers against the detrimental effects of exclusion. Indeed, the literature emphasizes that leadership practices play a fundamental role in shaping teachers' trust in their leaders [[66](#Tschannen)]. This finding is consistent with a broad body of literature indicating that various positive leadership approaches enhance organizational commitment. For example, person-centered leadership styles [[67](#Liqiu)], ethical leadership behaviors [[68](#Balıkçı)], and supportive climates where principals build teacher trust by supporting students' psychological needs [[69](#Adams)] have been shown to increase teacher commitment. Through these practices, principals create a supportive work environment where teachers feel valued and respected [[70](#Minott)].

The partial mediating role of othering and isolation can be explained by the Psychological Exclusion Model [30]. This model emphasizes that belonging is a fundamental human need. Findings reveal that the perception of being trusted by the principal provides a strong signal of inclusion for teachers and serves as a functional antidote to the professional exclusion frequently experienced by physical education teachers [[71](#Bollwerk)]. This finding is significant, as the literature consistently highlights that physical education teachers are often isolated from the school's central decision-making mechanisms and peer networks, which increases their experiences of professional loneliness and “being left alone” [[72](#Spicer)]. Moreover, this isolation is not without consequences; when perceived organizational support is low, it can weaken job satisfaction by increasing emotional exhaustion, a dynamic observed among physical education academics [[73](#Kinder)]. Therefore, perceived trust in the school principal serves a protective function not only at the individual level but also in terms of professional solidarity and job satisfaction.

Considering Turkey's collectivist cultural structure, the findings gain even greater significance. In collectivist contexts, the social trust signal conveyed by the leader is perceived more intensely; this is not only a matter of professional courtesy but also a strong confirmation of the teacher's position within the group. This perspective is supported by studies that approach leadership through a cultural lens. Indeed, Özdemir et al. (2023) [[74](#Özdemir)] demonstrate how cultural values, such as the avoidance of uncertainty, shape the role of leadership effects on teachers' perceptions of collective efficacy. In this context, it can be said that trust is a culturally intensified social signal. Furthermore, studies on the function of trust and friendship networks in teachers' professional relationships in Turkey reveal that these trust networks strengthen teachers' social belonging and cooperation within the school [[75](#Özenç)]. Conversely, a lack of trust can be interpreted as a deeper form of social exclusion, which further highlights the relationships observed in our study.

However, viewing perceived trust alone as a sufficient solution would be reductive. The findings require a more nuanced assessment. Three key points emerge in the literature: First, perceived trust may not alone compensate for the adverse effects of systemic issues such as role stressors [[36](#Sakallı36)]. Second, the impact of trust signals is limited to the leader's perceived competence and reliability; the leader must continuously earn trust to be sustainable [[76](#Batsaikhan)]. Third, excessive or unconditional trust can lead to adverse outcomes just as much as insufficient trust, so trust may need to be maintained at an optimal level [[77](#Butler)].

**Theoretical implications**

The main theoretical contribution of this research is its emphasis on the importance of the perceptual dimension in organizational dynamics. The methodological alignment of the construct (perceived trust) with the data source (teachers) provides a more robust framework for leadership research. Furthermore, it deepens Social Change Theory in an educational context by positioning perceived trust as a fundamental socio-emotional reward that triggers reciprocity. It also operationalizes the Psychological Exclusion Model by demonstrating that reduced marginalization is a critical mechanism in mediating the positive effects of perceived leadership support on commitment. Thus, it is revealed that the essential role of employee perception can explain inconsistencies in the literature regarding direct links between leadership behaviors and commitment.

**Practical implications**

The findings provide important insights for education leaders and policymakers. Leadership programs should focus not only on building trust but also on effectively communicating that trust to teachers. Transparent communication, collaborative decision-making, and systematic recognition of contributions are key in this context. These practices create an inclusive school culture where all teachers—especially those at high risk of marginalization—feel valued and supported. To reduce marginalization, it is a critical strategy for school administrations to ensure that physical education teachers are included in institutional-level decision-making mechanisms. Such practices not only increase teacher commitment but also strengthen the institutional integrity of the school.

**Limitations and future studies**

The findings of this study should be interpreted in light of several key limitations.

First, the study's generalizability is constrained by its sampling methodology. The use of a non-probability convenience sampling method means that the sample is not fully representative of all physical education teachers in Turkey, which limits the external validity of the results. Furthermore, the sample is characterized by a notable gender imbalance, with a significantly higher proportion of male participants (71.3%). While this may, to some extent, reflect the historically male-dominated nature of the physical education specialization in Turkey, it cannot be ruled out that this disparity is also an artifact of the sampling method. This imbalance restricts the generalizability of the findings, particularly concerning the experiences of female teachers. The voluntary nature of participation may also have introduced a selection bias, as teachers with stronger opinions or different motivation levels might have been more inclined to participate.

Second, the study's cross-sectional design reveals associations between variables but does not allow for the establishment of causal relationships. Due to the single point-in-time data collection, it is not possible to determine the causal direction of the observed relationships. For instance, while our model posits that perceived trust influences commitment, it is also plausible that teachers with higher pre-existing organizational commitment are more likely to perceive their principals' actions in a positive light, thereby fostering greater trust.

Future research should be designed to address these limitations. Replicating these findings with larger, more balanced, and representative samples drawn through stratified or random sampling techniques would be a valuable step to enhance generalizability. To clarify the causal pathways, longitudinal studies that follow the same teachers over time are essential. Furthermore, comparative studies across different cultural contexts are needed to test the generalizability of the proposed model. Finally, employing more advanced methods, such as structural interventions or experimental designs, would allow for a more rigorous evaluation of the long-term effects of trust-building interventions and could make significant contributions to the literature.

**Conclusion**

This study demonstrates that teachers’ perceived trust from their principals is a central determinant of their organizational commitment, both directly and indirectly. The results reveal that while teachers’ perceptions of principal trust strongly and positively predict their commitment, these perceptions also reduce feelings of marginalization and isolation, which in turn partially mediate the relationship between principal trust and commitment. These findings underscore the importance of how trust is perceived by teachers, highlighting that strengthening perceptions of trust can enhance their sense of belonging and professional value while mitigating marginalization and isolation within schools.

**Abbreviations**

X2/(CMIN): Ki-kare Minimum discrepancy

Df= Degree of freedom

GFI: Goodness of Fit İndex

AGFI: Adjustment Goodness of Fit İndex

CFI: Comparative Fit İndex

RMSEA: Root Mean Square Error of Approximation

SRMR: Standardized Root Mean Square Residual

CFA: Confirmatory Factor Analysis

AVE: Average Variance Extracted

CR: Composite Reliability

VIF: Variance Inflation Factor

SPTT: School Principals' Trust in Teachers

OC: Organizational Commitment

MI: Marginalization and Isolation

LLCI: Lower-Level Confidence Interval

ULCI: Upper-Level Confidence Interval

**Declarations**

**Ethical Approval and Procedure**

The data of the study were collected after the decision of Erzincan Binali Yıldırım University Human Research, Health and Sports Sciences Ethics Committee No. 10/04 in session 10 dated 25.10.2024 and after the informed consent of the participants was obtained. Participants were informed that they could withdraw from the study at any time. All procedures performed in this study comply with the 1964 Helsinki Declaration and ethical standards.

**Consent for publication**

Respondents were assured that participation in this research was strictly voluntary and that they could withdraw at any stage without any possible consequences. It was also assured that the responses would be kept confidential and used for research only. They consented to the completion and submission of the survey.

**Availability of data and material**

The datasets generated and/or analyzed during the current study are not publicly available due to confidentiality and ethical restrictions but are available from the corresponding author on reasonable request.

**Competing interests**

The authors declare no competing interests.

**Funding**

No funding has been received for conducting this study.

**Author contributions**

Conceptualization, HBE and AYM; formal analysis, ES; investigation, OKE and HBE; theoretical framework and hypotheses development, HBE and AYM; data collection, ES and OKE; methodology and data analysis, ES and OKE; supervision, OKE; validation, ES; writing—original draft, AYM; writing—review and editing, HBE, AYM, ES and OKE

**Acknowledgements**

Not applicable.

**References**

1. Cengiz Y, Demirdağ S. Analysis of master's theses and articles on the subject of trust in the manager. Asya Studies. 2024;8(29):15-28. <https://doi.org/10.31455/asya.1496710>.
2. Quines L, Relacion M. The mediating effect of school climate on the relationship between teacher communication behavior and student engagement. European Journal of Education Studies. 2022;9(11). <https://doi.org/10.46827/ejes.v9i11.4521>.
3. Ukuşlu G, Ukuşlu H, Aytekin S, Aytekin U. Teachers' views and expectations on school climate. National Education Journal. 2023;3(8):1090-1119. https://doi.org/[10.5281/zenodo.8312697](https://doi.org/10.5281/zenodo.8312697).
4. Lenz A, Rocha L, Aras Y. Measuring school climate: A systematic review of initial development and validation studies. International Journal for the Advancement of Counselling. 2020;43:48-62. <https://doi.org/10.1007/s10447-020-09415-9>.
5. Zheng X, Yin H, Liu Y. The relationship between distributed leadership and teacher efficacy in China: The mediation of satisfaction and trust. The Asia-Pacific Education Researcher. 2019;28(6):509-518.
6. Martinsone B, Žydžiūnaite V. Teachers’ contributions to the school climate and using empathy at work: Implications from qualitative research in two European countries. Frontiers in Psychology. 2023;14:1160546. <https://doi.org/10.3389/fpsyg.2023.1160546>.
7. Sarumaha YA, Firdaus DR, Moridu I. The application of artificial bee colony algorithm to optimizing vehicle routes problem. JISTE (Journal of Information System, Technology and Engineering). 2023;1(1):11-15. <https://doi.org/10.61487/jiste.v1i1.9>
8. Ramli A, Mundzir M, Suprianto G, Ashadi F, Arifudin O. Analysis of the influence of organizational commitment on work discipline of public high school teachers. Journal on Education. 2024;6(2):12927-12934. <https://doi.org/10.31004/joe.v6i2.5142>.
9. Yuwono B. The effect of commitment and environmental uncertainty on the relationship between budget participation and budget gap. Journal of Business and Accounting. 2023;1(1).
10. Al-Refaei AAA, Ali HBM, Ateeq AA, Alzoraiki M. An integrated mediating and moderating model to improve service quality through job involvement, job satisfaction, and organizational commitment. Sustainability. 2023;15(10): 7978. <https://doi.org/10.3390/su15107978>.
11. Richards K, Gaudreault KL, Starck JR, Mays Woods A. Physical education teachers’ perceptions of perceived mattering and marginalization. Physical Education and Sport Pedagogy. 2018;23(4): 445-459. <https://doi.org/10.1080/17408989.2018.1455820>.
12. Gaudreault KL, Richards K, Mays Woods A. Initial validation of the physical education marginalization and isolation survey (PE-MAIS). Measurement in Physical Education and Exercise Science. 2017;21(2):69-82. <https://doi.org/10.1080/1091367X.2016.1257994>.
13. Shoval E, Erlich I, Fejgin N. Mapping and interpreting novice physical education teachers' self-perceptions of strengths and difficulties. Physical Education and Sport Pedagogy. 2010;15(1):85-101. <https://doi.org/10.1080/17408980902731350>.
14. Flinders, D. J. (1988). Teacher isolation and the new reform. Journal of Curriculum & Supervision, 4(1).
15. Lortie D. School-teacher: a sociological study. Chicago: University of Chicago Press; 1975.
16. Gaudreault KL, Woods AM. The effects of achieved national board for professional teaching standards certification on the marginality of physical education teachers. The Teacher Educator. 2012;47(4):283-301. <https://doi.org/10.1080/08878730.2012.707760>.
17. Walters W, Robinson D, Barber W, Spicer C. (2024). The physical, social, and professional isolation of physical education teachers. Curriculum Studies in Health and Physical Education. 2024;1–21. <https://doi.org/10.1080/25742981.2024.2311116>.
18. Gök EBÇ. Organizational results of secondary school teachers' formal and natural group relationships in schools. Bartın University Journal of Faculty of Education. 2022;11(1):199-221.
19. Razeto PA, Cristina A. La confianza interpersonal entre profesores y directores de escuelas: un pilar fundamental para la implementación de políticas y programas educativos. 2009;15:57-81. <https://doi.org/doi:10.29344/07180772.15.628>
20. Dursun E. The relation between organizational trust, organizational support and organizational commitment. African Journal of Business Management. 2015:9(4);134-156. https://doi.org/10.5897/AJBM2014.7623
21. Babaoglan E. The predictive power of organizational trust to organizational commitment in elementary and high school teachers. The Anthropologist. 2016:24(1);83-89. <https://doi.org/10.1080/09720073.2016.11891992>
22. Fante C, Palermo S, Auriemma V, Rosalba M. Social inclusion and exclusion: how evolution changes our relational and social brain. In Evolutionary Psychology Meets Social Neuroscience. 2021. <https://doi.org/10.5772/intechopen.99916>
23. Munguia KA. The relationship among social and psychological inclusion, marginalized group membership, and student outcomes [doctoral dissertation]. Orange (CA): Chapman University; 2022.
24. Hutchison P, Ewens E. Social inclusion and the role of psychologists. In: Fleury-Bahi G, Levasseur R, Navarro O, editors. Handbook of social inclusion: research and practices in health and social sciences. Cham: Springer International Publishing; 2021. 1–17.
25. Tsai C-L, Ju J, Chen Z. The mediating role of prosocial and antisocial behaviors between team trust and sport commitment in college basketball players. European Journal of Sport Science. 2021:22(9);1418-1425. <https://doi.org/10.1080/17461391.2021.1973571>
26. Ulaş M, Şenel E. The relationship between commitment to teaching, teacher efficacy, marginalisation and isolation: a study on physical education teachers. Cypriot Journal of Educational Sciences. 2020;15(6):1439-1453. <https://doi.org/10.18844/CJES.v15i6.5217>
27. Nazir S, Qun W, Hui L, Shafi A. Influence of social exchange relationships on affective commitment and innovative behavior: Role of perceived organizational support. Sustainability. 2018;10(12):4418. <https://doi.org/10.3390/su10124418>.
28. Chow IHS. The mechanism underlying the empowering leadership-creativity relationship. Leadership & Organization Development Journal. 2018;39(2):202-217. <https://doi.org/10.1108/LODJ-03-2016-0060>.
29. Graen GB, Uhl-Bien M. Relationship-based approach to leadership: Development of leader-member exchange (LMX) theory of leadership over 25 years: Applying a multi-level multi-domain perspective. The Leadership Quarterly 1995;6(2):219–247. <https://doi.org/10.1016/1048-9843(95)90036-5>
30. Williams KD. (2007). Ostracism. Annual Review of Psychology. 2007;58:425-452. <https://doi.org/10.1146/annurev.psych.58.110405.085641>.
31. Yazgı K, Fazilet A, Kaş E, Sevim E. The effects of educational administrators in schools and institutions on teacher motivation. Eurasian Education & Literature Journal. 2024;(19):71-88.
32. Somech A. Directive versus participative leadership: Two complementary approaches to managing school effectiveness. Educational Administration Quarterly. 2005;41(5):777-800. <https://doi.org/10.1177/0013161X05279448>.
33. Dong Y, Bartol KM, Zhang ZX, Li C. Enhancing employee creativity via individual skill development and team knowledge sharing: influences of dual-focused transformational leadership. Journal of Organizational Behavior. 2017;38(3):439-58. <https://doi.org/10.1002/job.2134>
34. Bellibas MS, Liu Y. Multilevel analysis of the relationship between principals’ perceived practices of instructional leadership and teachers’ self-efficacy perceptions. Journal of Educational Administration. 2017;55(1):49-69. <https://doi.org/10.1108/JEA-12-2015-0116>.
35. Organista N, Mazur Z, Fletcher T. (2024). Towards precarity? The occupational  situation of physical education teachers in Poland. European Physical Education Review. 2024;0(0). <https://doi.org/10.1177/1356336X241269654>
36. Sakallı D, Şenel E. The role of marginalisation and role stressors in physical education teachers’ perceived mattering. European Physical Education Review. 2024;31(1):3- 17. <https://doi.org/10.1177/1356336X241245044>
37. Canlı S, Demirtaş H, Özer N. School administrators’ tendencies towards change. Elementary Education Online. 2015;14(2):634-646.
38. Sevinç YS, Sağlam AÇ. Relationship between teachers’ opinions and organizational confidence levels of communication skills of school administrators. Usak University Journal of Social Sciences. 2018;11(1):56-65.
39. Nacar D, Demirtaş Z. Teachers 'views on qualifications of school administrators. The Journal of Kesit Academy. 2017;(11):443-456.
40. Sarwar U, Tariq R, Yong OZ. Principals' leadership styles and its impact on teachers' performance at college level. Frontiers in Psychology. 2022;13:1-8. <https://doi.org/10.3389/fpsyg.2022.919693>.
41. Leithwood K, Harris A, Hopkins D. Seven strong claims about successful school leadership revisited. School Leadership & Management. 2020;40(1):5-22. <https://doi.org/10.1080/13632430701800060>.
42. Akbaşlı S, Diş O. Competencies of school managers as a leader according to teachers' views. International Journal of Leadership Studies: Theory and Practice. 2019;2(2):86-102.
43. Musah MB. Head teacher-teacher relationship and its effect on teacher's trust in Malaysian high performing schools. International Journal of Educational Management. 2018;32(3):479-497. <https://doi.org/10.1108/IJEM-10-2016-0204>.
44. Kırkıç KA, Balcı TD. Organizational commitment levels of preschool teachers and administrators' leadership styles. Problems of Education in the 21st Century. 2021;79(2):207. <https://doi.org/10.33225/pec/21.79.207>.
45. Yao H, Ma L. Improving teacher career satisfaction through distributed leadership in China: The parallel mediation of teacher empowerment and organizational commitment. International Journal of Educational Development. 2024;104:102960. <https://doi.org/10.1016/j.ijedudev.2023.102960>.
46. Liu L, Liu P, Yang H, Yao H, Thien LM. The relationship between distributed leadership and teacher well-being: The mediating roles of organisational trust. Educational Management Administration & Leadership. 2024;52(4):837-853. <https://doi.org/10.1177/17411432221113683>.
47. Parker MB, Curtner-Smith MD. Sport education: A panacea for hegemonic masculinity in physical education or more of the same? Sport, Education and Society. 2012;17(4):479-496. <https://doi.org/10.1080/13573322.2011.608945>.
48. Stroot SA, Ko Bomna. Induction of beginning physical educators into the school setting. In: The Handbook of Physical Education. 2006. p. 425.
49. Şenel E, Yıldız M, Ulaş M, Tamer K. The validity and reliability study of the Turkish form of physical education marginalization and isolation scale (PE-MAIS). Gazi Journal of Physical Education and Sport Sciences. 2019;24(3):155-166.
50. Chen LF, Khuangga DL. Configurational paths of employee reactions to corporate social responsibility: An organizational justice perspective. Corporate Social Responsibility and Environmental Management. 2021;28(1):389-403. <https://doi.org/10.1002/csr.2056>.
51. Christensen LB, Johnson RB, Turner LA. Araştırma yöntemleri desen ve analiz. Ahmet Aypay, çev. ed. Ankara: Anı Yayıncılık; 2015.
52. Gürbüz S. Aracı ve düzenleyici etki analizleri. Ankara: Seçkin Yayıncılık; 2021.
53. Cohen S, Manion L, Morrison K. Research methods in education. London: Routledge; 2011.
54. Richard FD, Bond CF, Stokes-Zoota JJ. One hundred years of social psychology quantitatively described. Review of General Psychology. 2003;7(4):331-363. <https://doi.org/10.1037/1089-2680.7.4.331>.
55. Canlı S, Demirtaş H, Özer N. The reliability and validity study of determining school principals’ trust in teachers scale - teacher form. Inonu University Journal of the Faculty of Education. 2017;18(3):103-117. <https://doi.org/10.17679/inuefd.310770>.
56. Üstüner M. Teachers’ organizational commitment scale: A validity and reliability study. Inonu University Journal of the Faculty of Education. 2009;10(1):1-18.
57. Kline RB. Principles and practice of structural equation modeling. 5th ed. New York (NY): Guilford Publications; 2023.
58. Hair JF, Black WC, Babin BJ, Anderson RE, Tatham RL. Multivariate data analysis. London: Pearson Education Limited; 2013.
59. Hayes AF. Introduction to mediation, moderation, and conditional process analysis: A regression based approach. New York, NY: Guilford Publications; 2022.
60. Hayes AF, Montoya AK, Rockwood NJ. The analysis of mechanisms and their contingencies: PROCESS versus structural equation modelling. Australasian Marketing Journal. 2017;25(1):76-81. <https://doi.org/10.1016/j.ausmj.2017.02.001>.
61. Preacher KJ, Hayes AF. Asymptotic and resampling strategies for assessing and comparing indirect effects in multiple mediator models. Behavior Research Methods. 2008;40(3):879-891. <https://doi.org/10.3758/BRM.40.3.879>.
62. Byrne BM. Structural equation modeling with AMOS. New York: Routledge; 2016.
63. Kalaycı Ş. SPSS Applied multivariate statistical techniques. Ankara: Dinamik Academy; 2018.
64. Savas AC, Kosker E, Demir S, Utar N. Teachers’ perception on the relationship between change leadership and organizational commitment. International Journal of Educational Methodology 2015;1(1):9–18. <https://doi.org/:10.12973/ijem.1.1.9>
65. Shen J, Ma X, Mansberger N, Wu H, Palmer LAB, Poppink S, Reeves PL. The relationship between growth in principal leadership and growth in school performance: The teacher perspective. Studies in Educational Evaluation 2021;70:101023. <https://doi.org/10.1016/J.STUEDUC.2021.101023>
66. Tschannen-Moran M. Leadership practices and why they matter: trust in leaders. 2023. <https://doi.org/10.1016/b978-0-12-818630-5.05029-6>
67. Liqiu Z. Person-oriented leadership style and organizational commitment in Chinese universities. Journal of Digitainability, Realism & Mastery (DREAM) 2022;1(4):102–111.
68. Balıkçı A. Examining the relationship between the ethical behaviours of school principals and organisational trust. Journal of Education for Life. 2022;36(3): 610-626 <https://doi.org/10.33308/26674874.2022363440>
69. Adams C, Adigun OB. Building a climate of faculty trust in students through principal support of student psychological needs. Journal of Educational Administration 2021;59(5):598–614. <https://doi.org/10.1108/JEA-08-2020-0188>
70. Minott MA. How head teachers can create a supportive workplace where teachers feel valued and respected. Management in Education 2024. <https://doi.org/10.1177/08920206241281621>
71. Bollwerk M, Wetzel E, Schlipphak B, Back MD. Who feels marginalized? A latent class analysis of perceived societal marginalization. Group Processes & Intergroup Relations. 2024;27(3):597–618. <https://doi.org/10.1177/13684302231182924>
72. Spicer C, Robinson DB. Alone in the Gym: A review of literature related to physical education teachers and isolation. Kinesiology Review. 2021. <https://doi.org/10.1123/KR.2020-0024>
73. Kinder CJ, Richards K, Trad AM, Woods AM, Graber KC. Perceived organizational support, marginalization, isolation, emotional exhaustion, and job satisfaction of PETE faculty members. European Physical Education Review. 2023. <https://doi.org/10.1177/1356336x231159636>
74. Özdemir S, Sezgin F, Kılınç AÇ, Polatcan M. A cultural lens to school leadership effects on teacher instructional practices: The mediation of teacher collective efficacy and the moderation of uncertainty avoidance. Educational Management Administration & Leadership 2025;53(3):515–535. <https://doi.org/10.1177/17411432231177536>
75. Özenç YY, Kalender S. Examining teachers' professional relationships, friendship, and trust networks via social network analysis. Uşak University Journal of Educational Research 2024;10(1):12–25. <https://doi.org/10.29065/usakead.1402654>
76. Batsaikhan M. Trust, trustworthiness, and business success: lab and field findings from entrepreneurs. Economic Inquiry. 2017;55(1)368-382. <https://doi.org/10.1111/ECIN.12359>
77. Butler JV, Giuliano P, Guiso L. The right amount of trust. Journal of the European Economic Association. 2016;14(5):1155-1180. <https://doi.org/10.1111/JEEA.12178>
